# On stage: the association between self-efficacy, flow, anxiety, and performers’ well-being in musical performance

**DOI:** 10.3389/fpsyg.2026.1859921

**Published:** 2026-07-08

**Authors:** Qihuan Chen, Yannan Liu, Xiaojing Sun, Li Pan

**Affiliations:** 1 School of Education and Foreign Languages, Wuhan Donghu University, Wuhan, China; 2 School of Humanities, Arts and Education, Shandong Xiehe University, Jinan, China; 3 School of Music, Inner Mongolia Minzu University, Tongliao, China

**Keywords:** flow, music performance anxiety, music performance self-efficacy, music performance well-being, PLS-SEM

## Abstract

**Introduction:**

In Chinese higher music education, performance-based assessments constitute a central component of students’ academic experience, and psychological factors are closely associated with performance outcomes. Although self-efficacy, flow, and anxiety have been widely discussed in music-related research, few studies have examined these variables together in relation to music performance well-being. Therefore, this study aims to examine the associations among music performance self-efficacy, flow, anxiety, and music performance well-being as experienced during music performance, and to investigate potential differences between vocal and instrumental music students.

**Methods:**

This cross-sectional study collected self-report questionnaire data from 986 Chinese university students majoring in vocal or instrumental music. Partial Least Squares Structural Equation Modeling was used to examine direct and indirect associations among the variables, and multi-group analysis was conducted to compare vocal and instrumental students.

**Results:**

The findings indicated that music performance self-efficacy was positively associated with well-being, with significant indirect associations involving flow and anxiety. Higher music performance self-efficacy was associated with higher flow and lower anxiety, and both higher flow and lower anxiety were associated with higher well-being, although the flow-related associations were relatively modest. Furthermore, the multi-group analysis revealed significant differences between vocal and instrumental students, suggesting domain-specific patterns in these associations.

**Discussion:**

These findings indicate that music performance well-being is closely associated with students’ performance-related confidence and anxiety. Music education should prioritize confidence-building and anxiety regulation when supporting students’ well-being in performance contexts. Support strategies should also consider domain-specific differences between vocal and instrumental students, as their psychological experiences may operate differently across performance modalities.

## 1 Introduction

Musical performance is not only an expression of art, but also a unique state of mind and emotion of the musician (Kaleńska-Rodzaj, 2025). Chinese higher music education places great importance on stage practice and stage competence, which demands university music students regularly perform in the classroom, examination recitals, and other types of stage work (Bao and Yu, 2025; He et al., 2024). Intense training and evaluation situations indicate that students’ psychological experiences are closely related to the nature of their learning experiences, their achievement outcomes, and their learning as artists (Okan and Usta, 2021). Building on this, examining the well-being of music students is crucial, as it not only demonstrates their psychological adjustment to the intensive training and performance requirements but also affects their motivation, involvement in learning and development of their musical skills.

Existing studies have tended to concentrate on subjective well-being, looking at musicians’ life satisfaction and their overall emotional experiences (MacDonald, 2013; Croom, 2015; Creech et al., 2013). This subjective well-being is often defined as multidimensional positive psychological conditions such as emotions, engagement, relationships, meaning, and achievement that capture their psychological health and optimal performance in music (Ascenso et al., 2018; Ascenso et al., 2017). However, music performance is characterized by a highly contextualized setting, public evaluation, and rapid fluctuations in emotional states (Nicholson et al., 2015; Guyon et al., 2020; Murphy et al., 2025), making it necessary to further examine subjective well-being specifically within the context of music performance. In the present study, music performance well-being (MPWB) is conceptualized as a performance-specific operationalization of well-being. Rather than proposing MPWB as an entirely new theoretical construct, this study uses the term to refer to performers’ positive psychological experiences and overall mental states during musical performance, stage practice, and related performance activities. Specifically, MPWB reflects positive affective experiences, psychological adaptation, and functional engagement within the performance context. In this sense, MPWB represents a performance-contextualized well-being that allows the present study to examine how self-efficacy, flow, and performance anxiety are associated with students’ well-being in authentic and evaluative music performance situations. Compared with general subjective well-being, this contextualized approach may provide a more targeted way to capture the psychological states of musicians during performance. It does not imply that MPWB constitutes a separate theoretical framework, but rather that well-being can be meaningfully examined in relation to the emotional, cognitive, and evaluative demands of music performance. Accordingly, the present study adopts MPWB as a performance-specific indicator of subjective well-being to better understand students’ psychological experiences in authentic and evaluative music performance contexts.

Various cognitive and emotional processes influence the psychological experience one undergoes during musical performance. Among them, the perceptions of the performers on their ability to perform have been cited as a significant factor in the outcome of performance (McPherson and McCormick, 2006). In line with the social cognitive theory (Bandura, 1997), the beliefs that people have on their abilities are central to their responses to their emotions and experiences associated with performance. It is particularly critical in the situation of higher music education in China, where students are often expected to rehearse recitals and other performance-related evaluations (Jiang and Tong, 2025). The concept of music performance self-efficacy (MPSE) as the confidence of performers in their skills to successfully prepare and deliver musical performances (Bersh, 2022; Zelenak, 2011) thus plays an important role in determining the way students manage their performance-related experiences of demanding situations and control their psychological experiences to achieve more desirable outcomes (McPherson and McCormick, 2006).

Performers’ beliefs in their musical abilities, or MPSE, are closely associated with their psychological experiences during performance (Zarza-Alzugaray et al., 2020). Higher MPSE is linked to greater engagement in the task, reflected in the experience of flow (FL), a state of deep concentration, immersion, and reduced self-consciousness (Csikszentmihalyi, 1990; Habe et al., 2019). Flow experiences have also been linked to positive psychological outcomes in music learning and performance contexts, suggesting that positive performance-related experiences may be relevant to students’ emotional and psychological states (Li, 2026). FL, in turn, is associated with variations in music performance anxiety (MPA), as task-focused immersion may coincide with reduced attention to external evaluation or potential mistakes during performance (Spahn et al., 2021). Observations suggest that the relationship between flow and MPA is dynamic. Anxiety may inhibit flow before performance, flow may become more salient during performance, and afterward, flow experiences may be associated with how anxiety is perceived and regulated (Spahn et al., 2021). These theoretically informed associations describe patterns among FL and MPA as in-task psychological states. How these patterns relate specifically to MPWB, the positive psychological functioning experienced in evaluative music performance situations, remains to be empirically examined.

In addition, this study considers two groups of music students, vocal and instrumental, representing different modes of training and performance, to provide a contextualized perspective on the formation of MPWB. These groups differ substantially in terms of bodily engagement, the structure of immediate feedback, and the degree of evaluative exposure (Burwell, 2006; Leman, 2016), which may be associated with variations in how MPSE relates to FL, MPA, and MPWB. Furthermore, differences in the performance medium could also be associated with variations in the role of the instrument as a mediating object, which could relate to performers’ degree of self-exposure and psychological processing in evaluative contexts (Kenny, 2011), and may correspond with differences in MPWB under performance pressure. Thus, the comparison between vocal and instrumental students presents a new and valuable perspective, elucidating the psychological associations of MPWB, in different ways of musical training and musical setting.

Thus, the focus of this study is on MPSE, FL, and MPA as they are each specifically defined on musical performance contexts, and their collective patterns of association with MPWB. Also, by comparing vocal and instrumental students as distinct groups, it investigates how different training and performance modalities relate to these psychological processes. Analyzing these factors in authentic performance contexts illuminates the contextual nature of MPWB and provides a lens to complement previous studies, which have mainly investigated general subjective well-being. In doing so, it provides a nuanced, group-sensitive understanding of psychological patterns related to performers’ well-being and extends the theoretical understanding of musicians’ experiences in evaluative performance environments.

## 2 Literature reviews

### 2.1 Definitions of key constructs in music performance contexts

#### 2.1.1 Music performance self-efficacy (MPSE)

Self-efficacy is founded on the social cognitive theory developed by Bandura, which describes self-efficacy as a belief held by an individual with regard to their capability to organize and perform the actions needed to reach a certain level of performance (Bandura, 1986). MPSE can be described as the assumption of a performer that he or she can competently fulfill the technical, expressive, and contextual requirements of a musical task and not his or her musical talent (McPherson and McCormick, 2006). MPSE reflects the performer’s confidence in their musical abilities, as well as their subjective perception of the ability to cope with challenges and pressure during practice, rehearsal, and public performance (Ritchie and Williamon, 2011).

#### 2.1.2 Flow (FL)

FL, originally defined by Csikszentmihalyi (1990), refers to a state of complete immersion and focus in an activity, where individuals are fully engaged, often losing track of time and self-awareness. This state is characterized by a perfect balance between the challenges of the task and the individual’s skill level, leading to an intrinsically rewarding experience (Csikszentmihalyi, 1990). In the context of music performance, FL refers to a psychological state of total absorption and deep engagement in performing, where musicians experience intense focused attention, a loss of self-conscious awareness, and a distorted sense of time, often resulting in enhanced performance quality and intrinsic enjoyment of the musical task (Moral-Bofill et al., 2023).

#### 2.1.3 Music performance anxiety (MPA)

Anxiety is generally described as an emotional reaction toward the perceived danger or uncertainty, and it is marked by feelings of nervousness, worry, fear, and unease (Spielberger et al., 1983). Although it is a natural response, it turns out to be a problem when it surpasses the ability of a person to manage it, which may result in such disorders like generalized anxiety disorder (Beck, 1976). MPA is defined as the anxiety experienced by the musicians before, during, and after a performance, which is normally accompanied by both emotional responses such as fear, nervousness, and self-doubt and physiological responses such as trembling, dry mouth, and sweat (Kenny, 2011).

#### 2.1.4 Music performance well-being (MPWB)

Subjective well-being is generally understood as individuals’ overall evaluation of their quality of life and psychological functioning, typically involving dimensions such as positive emotions, engagement, meaning, relationships, and accomplishment (Diener, 1984; Seligman, 2011). In the present study, MPWB is conceptualized as a form of performance-contextualized subjective well-being. It should be noted that MPWB is not proposed as an entirely new construct developed exclusively for the music performance context. Rather, it refers to the contextual expression of subjective well-being within music performance and related activities. Specifically, MPWB describes the positive psychological experiences and overall mental states that individuals experience during musical performance, rehearsal, stage practice, and other performance-related activities. In line with the broader understanding of subjective well-being, MPWB reflects performers’ positive affective experiences, psychological adaptation, and functional engagement within the performance context.

### 2.2 Theoretical associations linking MPSE, FL, MPA, and MPWB

According to social cognitive theory, individuals’ psychological and behavioral outcomes are shaped through the reciprocal interactions among personal factors, behavior, and the environment (Bandura, 1986, 1997). Within this framework, self-efficacy represents a central personal belief that is closely related to how individuals interpret situational demands, regulate emotional responses, mobilize effort, and select coping behaviors when facing challenging tasks (Bandura, 1997). In other words, self-efficacy is associated with whether individuals appraise a demanding situation as a manageable challenge or as an uncontrollable threat. This theoretical perspective is particularly relevant to music performance, which is characterized by high task demands, public evaluation, and considerable psychological pressure (Papageorgi et al., 2013; Kenny, 2011). In such a context, MPSE does not merely reflect students’ general confidence in their musical ability. Rather, it represents their belief that they can successfully prepare for and complete a performance task, express musical ideas effectively, and cope with the technical, emotional, and evaluative demands of the performance situation (Ritchie and Williamon, 2011). From this perspective, higher MPSE may be associated with perceiving performance tasks as more controllable and achievable. This positive appraisal may be related to a stronger sense of competence, personal control, and psychological readiness during performance, as well as to a lower tendency to experience the performance situation as threatening. Accordingly, high-MPSE students may be more likely to report confidence, persistence, and positive emotional involvement during performance, which are important foundations for adaptive psychological functioning in performance contexts (McPherson and McCormick, 2006). These processes provide a theoretical basis for linking MPSE to MPWB. Since MPSE is associated with perceived competence, control, and positive task engagement, it may be related to more favorable performance-related psychological experiences. This argument is consistent with the view that competence, effective engagement, and positive psychological functioning are closely associated with well-being (Ryan and Deci, 2000). Therefore, MPSE can be expected to positively relate to students’ MPWB.

Flow theory emphasizes that individuals are more likely to enter a state of deep concentration and absorption when they perceive a balance between their own abilities and the challenges of the task (Csikszentmihalyi, 1990). In such a state, individuals often experience a heightened sense of control, smooth involvement, and intrinsic enjoyment during the activity (Csikszentmihalyi, 1990, 2000). In the context of music performance, performers’ experience of FL may be related not only to the objective difficulty of the performance task, but also to how they evaluate their own ability to meet its technical, expressive, and emotional demands (McPherson and McCormick, 2006). From this perspective, MPSE may be one psychological factor associated with the emergence of FL. Performers with higher MPSE are more likely to perceive performance challenges as manageable and achievable (McPherson and McCormick, 2006; Ritchie and Williamon, 2011), which may be associated with a stronger perceived balance between task demands and personal competence, a key antecedent of FL experience (Fong et al., 2015). This perception may be related to fewer concerns about insufficient ability or possible failure, as well as greater psychological involvement in musical expression, sound control, emotional communication, and real-time performance engagement (Moral-Bofill et al., 2023). In this context, performers may be more likely to report focused attention, immersion, control, and enjoyment during performance, which are central features of FL in musical contexts (Moral-Bofill et al., 2023; Spahn et al., 2021). FL may therefore be theoretically relevant to the association between MPSE and MPWB as a positive experiential component of music performance. When performers are deeply absorbed in musical performance, their attention is directed toward the musical task itself rather than toward self-doubt or external evaluation (Chirico et al., 2015; Lamont, 2012). This task-focused involvement is closely related to positive emotional experiences, perceived control, and adaptive psychological functioning during performance (Loepthien and Leipold, 2022). These experiences are closely aligned with MPWB, which emphasizes positive psychological states, emotional adaptation, and functional engagement within the performance context. Accordingly, FL may be understood as one possible experiential correlate linking MPSE with concentration, immersion, control, enjoyment, and more favorable MPWB experiences in music performance.

Gross’s theory of emotion regulation emphasizes that emotional responses are not determined solely by external events, but are also shaped by processes such as situation appraisal, attentional deployment, cognitive reappraisal, and response modulation (Gross, 1998, 2015). From this perspective, MPA should not be understood simply as a general state of nervousness. Rather, it reflects performers’ sustained worries and emotional arousal in evaluative performance situations, particularly concerns about failure, mistakes, negative evaluation, and uncontrollable outcomes (Kenny, 2011). Therefore, MPA not only indicates the intensity of anxiety during performance, but also reflects performers’ subjective perception of situational threat, insufficient personal competence, and the possible consequences of failure. In music performance contexts, MPSE may be closely related to these appraisal and regulation processes. Performers with higher MPSE are more likely to believe that they can cope with the technical demands, expressive requirements, and evaluative pressure involved in public performances, examinations, or juried assessments (Zelenak, 2011; Hu, 2024). Accordingly, higher MPSE may be associated with interpreting performance tasks as more controllable challenges rather than overwhelming threats (González et al., 2018). This belief in personal capability may be associated with lower expectations of failure, fewer concerns about negative evaluation, and a weaker sense of uncontrollability, all of which are closely related to lower levels of MPA. MPA may further represent a negative emotional pathway linking MPSE and MPWB. According to attentional control theory, anxiety weakens goal-directed attentional control and increases individuals’ attention to threat-related stimuli (Eysenck et al., 2007). In musical performance, higher MPA may be associated with greater cognitive and attentional load, with students’ attention more strongly oriented toward fear of mistakes, negative evaluation, and loss of control rather than toward the musical task itself (Kenny, 2011; Papageorgi et al., 2013). By contrast, lower levels of MPA may be associated with less anxiety-related cognitive interference and greater psychological availability for musical expression, sound control, emotional communication, and task engagement (Papageorgi et al., 2013; Yoshie et al., 2009). This pattern may be related to more positive, adaptive, and psychologically rewarding performance experiences, which are closely aligned with MPWB. Therefore, MPA can be understood as a negative emotional pathway through which MPSE is associated with MPWB.

Previous studies have generally indicated a negative relationship between flow (FL) and MPA. Cohen and Bodner (2021) found that higher levels of FL are usually accompanied by lower levels of MPA, suggesting that deep absorption and intense concentration are closely related to lower anxiety experiences during music performance. Furthermore, Spahn et al. (2021) examined the relationship between FL and MPA from a temporal dynamic perspective and argued that this relationship is not static across different stages of stage performance. Specifically, higher anxiety before performance may be associated with difficulty entering FL, whereas during the actual performance, immersion in the musical activity may coincide with higher FL and lower MPA. This finding suggests that FL and MPA may interact dynamically within the performance process. FL is a task-centered state of intense concentration, characterized by highly focused attention, reduced self-consciousness, and decreased concern with external evaluation (Csikszentmihalyi, 1990, 2000). In music performance contexts, when performers enter a state of FL, their cognitive resources are more likely to be directed toward the musical task itself rather than toward possible failure, mistakes, or external judgment (Csikszentmihalyi, 1990; Jackson and Marsh, 1996; Habe et al., 2019). This task-focused attentional involvement may be associated with lower anxiety-related cognitive interference and threat-monitoring processes (Kenny, 2011; Spahn et al., 2021; Steptoe and Fidler, 1987). Therefore, during actual performance, FL may be theoretically positioned before MPA in the proposed model because task-focused immersion is closely related to lower anxiety-related cognitive interference. However, this ordering should be understood as a theoretically informed model specification rather than evidence of temporal or causal precedence. Although the present study positions FL before MPA in the main model, the reverse ordering between MPA and FL is also theoretically plausible. MPA may be associated with difficulty entering FL, particularly when performers report stronger self-focused monitoring and greater sensitivity to mistakes, failure, and external evaluation. To address this theoretical possibility, the present study further specified MPSE → MPA → FL → MPWB as an alternative model in Supplementary material. Nevertheless, MPSE → FL → MPA → MPWB was retained as the primary model in the main text because this study emphasizes performers’ psychological experiences during actual music performance, where task-focused immersion and anxiety-related cognitive interference may be closely interrelated.

### 2.3 Hypotheses development

#### 2.3.1 Music performance self-efficacy

Although the relevance of self-efficacy in the overall experiences and psychological performance of musicians has been well-established, its particular association with psychological experiences in live performance situations has been under-researched. Su et al. (2024) stress that MPSE plays a vital role in promoting FL in music education, and this correlation shows that the belief of a music student in their abilities is able to contribute greatly to their engagement and general performance. Spahn et al. (2021) discovered that increased MPSE is a key predictor of FL proneness in singers, indicating that higher confidence in their talent is associated with deeper engagement and absorption in performance. The high MPSE musicians tend to be more persistent in tasks and perform effectively to alleviate MPA (González et al., 2018). High-MPSE performers tend to report better emotion regulation, remain composed under pressure, and recover more quickly from mistakes, which is associated with lower levels of music performance anxiety (Ou and Qin, 2025). Anand and Kumar (2013) found that self-efficacy was positively related to the subjective well-being of performing artists in India. In a similar manner, Kausar and Ahmad (2021) established that among performing arts students, self-efficacy was positively correlated with psychological well-being significantly. Thus, the hypothesis below was set.

*H1*: MPSE is positively associated with FL.

*H2*: MPSE is negatively associated with MPA.

*H3*: MPSE is positively associated with MPWB.

#### 2.3.2 Flow

Previous research has reported associations with various aspects of musicians’ psychological experiences, including MPA and subjective well-being. Cohen and Bodner (2021) found a negative relationship between FL and MPA, suggesting that higher FL is associated with lower MPA and that flow-related engagement is relevant to musicians’ anxiety experiences. Spahn et al. (2021) also reported a negative correlation between FL and MPA, indicating that complete engagement and focus during performance are associated with more adaptive anxiety-related experiences among musicians. Loepthien and Leipold (2022) found that FL during music is positively related to subjective well-being, and that this relationship is stronger when participants have a flexible self-concept. This suggests that FL is linked with overall well-being and may reflect adaptive psychological functioning in musical contexts. Similarly, Habe et al. (2021) found that FL is positively related to subjective well-being among music students, with flow experiences often linked to greater life satisfaction and emotional well-being. Based on these findings, the following hypotheses are proposed.

*H4*: FL is negatively associated with MPA.

*H5*: FL is positively associated with MPWB.

#### 2.3.3 Music performance anxiety

MPA has been found to be strongly related to performance and well-being, particularly in high-stress environments. Bao and Yu (2025) found that MPA is negatively related to well-being, indicating that higher levels of anxiety are associated with lower emotional well-being among music students. Matei and Ginsborg (2017) reported that MPA shows a connection with musicians’ well-being, with higher levels of anxiety tending to coincide with lower emotional health and reduced satisfaction with performance. According to Barros et al. (2024), MPA is linked to adverse psychological outcomes, and coping mechanisms tend to coincide with lower levels of MPA and higher levels of well-being among music students. Therefore, the hypothesis below was presented.

*H6*: MPA is negatively associated with MPWB.

**Figure 1:**
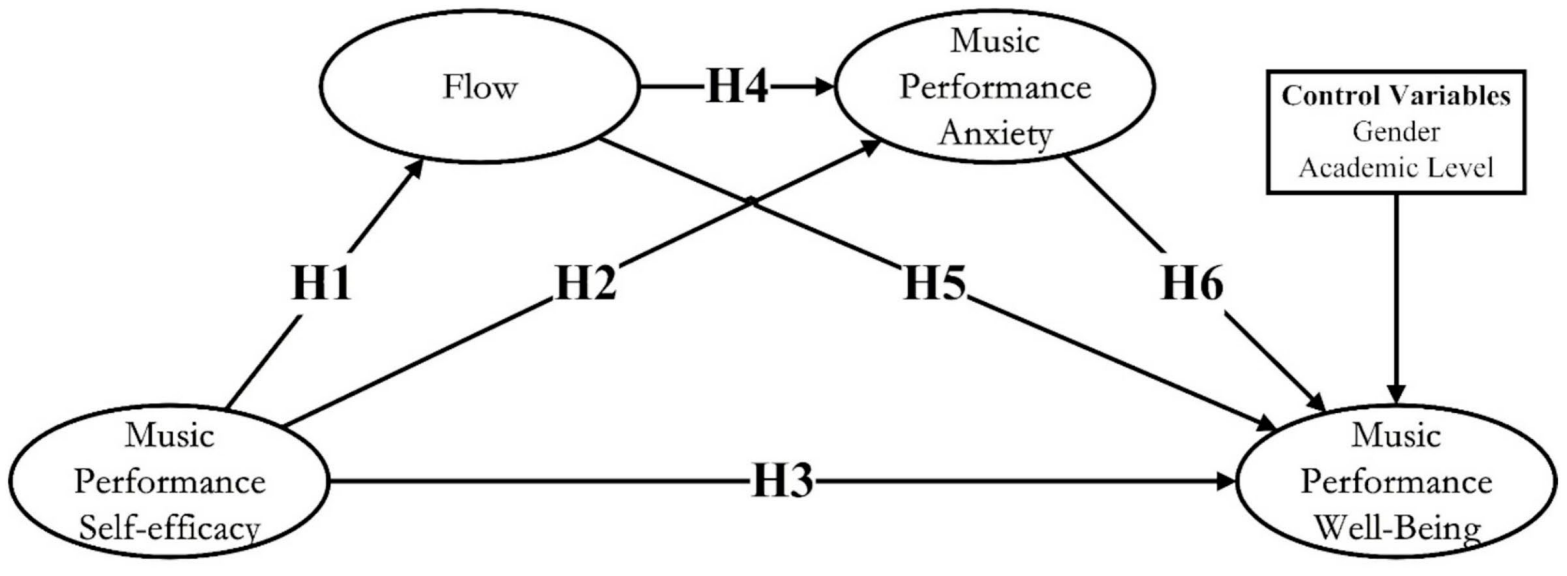
Conceptual framework.

Figure 1 illustrates the conceptual framework of this study, based on the hypotheses proposed above, with gender and academic level as control variables.

### 2.4 Theoretical rationale for group differences: vocal vs. instrumental students

Vocal and instrumental students exhibit substantial differences in performance modalities, which may have important implications for their psychological experiences and the pathways among relevant psychological variables. Li and Wang (2026) emphasized that music learning is highly context-specific, particularly in relation to embodied performance, real-time feedback, and collaborative coordination. Therefore, different forms of musical training, such as vocal and instrumental training, may require different types of pedagogical and psychological support. Embodied cognition provides a useful theoretical perspective for understanding these group differences. From this perspective, cognition does not occur solely within the brain, but is shaped through the dynamic interactions among the body, perception, action, and environment (Leman, 2016). Music performance is a highly embodied activity, and different performance media and forms of bodily involvement may be associated with performers’ emotion regulation, attentional control, and self-perception. Although both vocal and instrumental performance are embodied musical activities, they differ markedly in how the body participates in cognition and performance.

In vocal performance, the body itself serves as the primary medium of sound production. Vocal sound is closely related to physiological processes such as breath regulation, laryngeal control, muscular tension, and bodily posture. Thus, the body is not only an expressive tool, but also directly involved in sound production, emotional experience, and cognitive processing (Geeves and Sutton, 2014; Jiang and Wu, 2025). Vocalists must continuously monitor their breathing, vocal changes, and bodily sensations, and adjust their vocal production and emotional expression in real time based on this internal feedback (Kochman et al., 2012). Because there is no external medium that buffers the performer from the audience, the voice, facial expressions, and bodily posture of vocalists are directly exposed to public observation. This direct exposure may intensify bodily self-awareness, self-monitoring, and sensitivity to external evaluation (Papageorgi et al., 2013; Wells, 1995). From an embodied cognition perspective, cognition, emotion, and bodily states may be more directly interconnected in vocal performance, and vocal students’ psychological experiences may be more closely related to bodily feedback and evaluative performance contexts.

By contrast, embodiment in instrumental performance is more strongly reflected in the dynamic integration between the body and the instrument. Although instrumental performance also relies on motor control, tactile feedback, and auditory regulation, the instrument functions as an external mediating tool that partially assumes the role of sound production and musical expression (Malloch and Wanderley, 2017). Through long-term training, instrumental performers gradually incorporate the instrument into their body schema, allowing the instrument to become an extension of their bodily action capabilities. In this process, cognition emerges through continuous interaction among the body, the instrument, and the performance environment (Wilson and Golonka, 2013). In this context, instrumental performers’ attentional resources may be more strongly directed toward motor precision, technical control, and coordination between the performer and the instrument, rather than toward direct bodily exposure (Keller, 2001; Mornell and Wulf, 2019). Compared with vocal performance, emotional and anxiety-related experiences in instrumental performance may be more closely associated with technical errors or failures in motor control than with heightened self-consciousness related to the direct evaluation of the body itself (Kenny, 2011).

Music students, both vocal and instrumental, typically engage in continuous training related to the development of their performance skills. However, the specific modes of training differ markedly between disciplines, and such differences may further shape students’ psychological experiences and the pathways among relevant psychological variables. Real-time internal bodily feedback is an important aspect of vocal instruction, encompassing breath control, resonance modulation, and emotional expression. It is characterized by the inseparability of the body and the instrument, and focuses the embodied perception and regulation of inner experience (Burwell, 2006; Leman, 2016). From the perspective of embodied cognition, vocal performance can be understood as a dynamic process in which bodily movements, emotional states, and cognitive control interact continuously, with sound quality closely related to ongoing bodily awareness and regulation (Heisel, 2015). Instrumental training, on the other hand, focuses on fine control of external instruments and the co-ordination of several joints, which is a very complex sensorimotor integration process. Stability in motor execution and consistency in musical output are closely related to performers’ continuous allocation of attention among auditory feedback, visual cues, and proprioceptive input (Watson, 2006). In this complex sensorimotor context, attentional regulation is regarded as an important component of skill acquisition and optimization of movement. In particular, an external focus of attention and sustained attentional allocation have been associated with motor coordination and performance outcomes (Song, 2019).

Taken together, from the perspective of embodied cognition, the differences between vocal and instrumental performance in bodily involvement, perceptual feedback mechanisms, and performer–environment interaction may be associated with distinct patterns in psychological processes such as emotion regulation, self-awareness, attentional control, and stress coping. This further suggests that the relationships among MPSE, FL, and MPA, as well as their associations with MPWB, may vary between vocal and instrumental students. This rationale also aligns with the study’s design, which compares vocal and instrumental students as distinct groups in the proposed model.

## 3 Methods

### 3.1 Research design

This study adopted a cross-sectional survey to explore the relationship between MPSE, FL, MPA, and MPWB in Chinese university students who major in music. The self-report questionnaire was administered between December 25, 2025, and March 3, 2026, and the samples were selected from four arts universities in China, based on their geographical diversity and a representation of a broad variety of music students at diverse developmental stages. A purposive and convenience sampling method was used to recruit the participants. Purposive sampling was used to target the participants who were eligible according to a set of inclusion criteria, which included Chinese university students, undergraduate and graduate students, and majors in music, both vocal and instrumental. They were then sampled conveniently to get participants who were readily available and willing to give their input to ensure that there was a practical and accessible sample to collect data.

The researchers collected 1,320 questionnaires. Once the questionnaires with missing values (199) and those with too similar responses to scale items (135) were eliminated, 986 appropriate questionnaires were left. The researchers divided the returned questionnaires according to the music major of the participants and divided them into instrumental (521) and vocal (465) participants. By following the requirements of the ethics review committee to maintain the privacy of the participants, only gender and academic level were taken as demographic information. Among the instrumental group, there were 89 males (17.1%) and 432 females (82.9%), with 425 undergraduates (81.6%) and 96 graduate students (18.4%). Among the vocal group, there were 122 males (26.2%) and 343 females (73.8%), with 374 undergraduates (80.4%) and 91 graduate students (19.6%).

### 3.2 Research instruments

The structured self-report questionnaire was used to gather data online. The questionnaire had four sections. This was in the form of a first part that comprised screening questions that were meant to ensure that all the participants were eligible to participate in this study, which, in this case, was that the participants had to be Chinese university students pursuing vocal or instrumental music (not music education). In the second part, there was an electronic informed consent form. This section enlightened the participants on the aim of the study, the kind of data gathered, the data analysis techniques, and the data storage and subsequent use. The participants who expressed their consent by choosing the option I agree to participate in this study could only move on to the rest of the questionnaire. The third section was used to gather demographic data, such as gender and current level of study. The fourth part included standard measurement scales that measured the key constructs of the study.

All scale items were measured using a five-point Likert scale ranging from 1 (strongly disagree) to 5 (strongly agree). Given that all participants were Chinese students, the original questionnaire was translated into Chinese by a professional translation agency. Subsequently, two Chinese professors specializing in English studies independently translated the Chinese version back into English. The back-translated versions were then carefully compared with the original instruments through repeated reviews to ensure semantic equivalence, conceptual consistency, and translation accuracy.

MPSE was measured using an adapted version of the scale developed by Ritchie and Williamon (2011), which consists of nine items. FL was assessed using an adapted version of the Flow State Scale developed by Jackson and Marsh (1996), which includes nine items. MPA was assessed using an adapted version of the scale proposed by Mazzarolo and Schubert (2022), comprising five items. MPWB was measured using an adapted five-item version of the scale developed by Topp et al. (2015), which was originally designed to assess general subjective well-being and therefore required modification to capture well-being specifically within the context of music performance. The core psychological dimensions (positive mood, calmness, energy, and interest) were preserved, and all items were reworded to reflect experiences during music performance. Content validity was evaluated by six professional musicians using the Content Validity Index, with all items achieving an I-CVI of 1.00 and a scale-level S-CVI of 1.00. Minor suggestions from two experts were incorporated after discussion with the guiding professor. The fully revised MPWB scale is presented in Supplementary Table S1. Before the formal distribution of the questionnaire, the researchers conducted a pilot test with 50 participants and found that the Cronbach’s alpha values for all constructs ranged from 0.765 to 0.821, indicating that the scale used in this study has adequate internal consistency and is suitable for official distribution.

### 3.3 Data analysis

Descriptive statistics were first performed by SPSS 26 to examine the basic characteristics of the data. Partial least squares structural equation modeling (PLS-SEM) was conducted by SmartPLS 4.1. PLS-SEM was selected over covariance-based SEM (CB-SEM) for several reasons. First, the primary aim of this study was to examine the predictive relationships among multiple psychological constructs rather than to confirm a well-established theoretical model (Hair and Alamer, 2022). Second, the proposed model is relatively complex, involving multiple latent variables, parallel mediators, and interaction effects, for which PLS-SEM provides greater analytical flexibility and robustness (Hair and Alamer, 2022). To examine the theoretical plausibility of alternative specifications, an alternative model with the ordering MPSE → MPA → FL → MPWB was specified. Comparisons between the original and alternative models, including direct effects, indirect (mediation) effects, *R*^2^, *f*^2^, and model fit indices, are provided in Supplementary Tables S1–S5. The primary distinction between the two models lies in the relative positioning of FL and MPA. In the alternative model, MPA is positioned as preceding FL, representing a plausible configuration in which anxiety and flow are associated in a different sequence within the performance context. Afterward, the researchers continued to use Bootstrap multi-group analysis to compare the model paths between vocal and instrumental music students, conducting a multi-group comparison to examine potential differences in the relationships between the constructs across these two groups.

## 4 Results

### 4.1 Measurement model testing

The Variance Inflation Factor (VIF) values for indicators ranged from 1.503 to 3.006, and for constructs, they ranged from 1.193 to 1.369, all below the threshold of 3.3 (Kock and Lynn, 2012), indicating no multicollinearity concerns. The Full VIF analysis for Common Method Bias (CMB) was conducted by extracting the latent variable scores from SmartPLS, and then calculating the VIF values in SPSS, which ranged from 1.364 to 2.905. These values are well below the threshold of 3.3, suggesting that CMB is not a significant concern in this study.

As shown in Table 1, the factor loadings of all items ranged from 0.719 to 0.887, indicating adequate indicator reliability, as all loadings exceeded the recommended threshold of 0.7. The internal consistency reliability was assessed by Cronbach’s alpha (CA) and composite reliability (CR). CA ranged from 0.830 to 0.921 and CR ranged from 0.880 to 0.941 respectively, which are both higher than the acceptable value of 0.7, showing good internal consistency. Convergent validity was measured by Average Variance Extracted (AVE) and found to range from 0.576 to 0.760. The convergent validity of all AVE values was higher than the threshold value of 0.5 (Fornell and Larcker, 1981).

**Table 1:** Convergent validity.

Constructs	Items	Loading	CA	CR	AVE
MPSE	MPSE1	0.727	0.908	0.924	0.576
	MPSE2	0.788			
	MPSE3	0.748			
	MPSE4	0.784			
	MPSE5	0.740			
	MPSE6	0.730			
	MPSE7	0.789			
	MPSE8	0.796			
	MPSE9	0.724			
FL	FL1	0.777	0.916	0.925	0.578
	FL2	0.774			
	FL3	0.743			
	FL4	0.733			
	FL5	0.736			
	FL6	0.757			
	FL7	0.753			
	FL8	0.791			
	FL9	0.775			
MPA	MPA1	0.754	0.830	0.880	0.596
	MPA2	0.719			
	MPA3	0.808			
	MPA4	0.787			
	MPA5	0.788			
MPWB	MPWB1	0.883	0.921	0.941	0.760
	MPWB2	0.870			
	MPWB3	0.887			
	MPWB4	0.852			
	MPWB5	0.868			

Discriminant validity was assessed using two methods: the Fornell-Larcker criterion and the heterotrait-monotrait ratio (HTMT). As shown in Table 2, the square root of the AVE for each construct was greater than the Pearson correlation coefficients between the constructs, indicating satisfactory discriminant validity according to the Fornell-Larcker criterion (Fornell and Larcker, 1981). The HTMT values ranged from 0.136 to 0.831, with 95% bias-corrected bootstrap confidence intervals based on 5,000 subsamples (Table 3). All HTMT values and their corresponding confidence intervals were below the threshold of 0.85, further supporting the discriminant validity of the constructs (Henseler et al., 2015).

**Table 2:** The Fornell-Larcker criterion.

Constructs	MPSE	FL	MPA	MPWB
MPSE	0.759			
FL	0.093	0.76		
MPA	−0.511	−0.121	0.772	
MPWB	0.676	0.28	−0.729	0.872

**Table 3:** HTMT (95% bias-corrected bootstrapping).

Path	HTMT	Sample mean (*M*)	2.50%	97.50%
MPA ↔ FL	0.137	0.143	0.107	0.187
MPSE ↔ FL	0.136	0.143	0.119	0.175
MPSE ↔ MPA	0.580	0.580	0.529	0.626
MPWB ↔ FL	0.253	0.254	0.195	0.313
MPWB ↔ MPA	0.831	0.830	0.812	0.846
MPWB ↔ MPSE	0.735	0.735	0.703	0.765

### 4.2 Structural model testing

The researchers performed hypothesis testing using SmartPLS with a bootstrapping procedure involving 5,000 resamples. As shown in Table 4, all hypotheses are supported. MPSE is positively associated with FL (*β* = 0.093) and MPWB (*β* = 0.404), and shows a negative association with MPA (*β* = −0.505). FL is positively associated with MPWB (*β* = 0.182) and shows a negative association with MPA (*β* = −0.075), while MPA is negatively associated with MPWB (*β* = −0.500). Gender (*β* = −0.018, *p* = 0.672) and academic level (*β* = 0.031, *p* = 0.503) do not show significant associations with MPWB, indicating that these control variables are not strongly related to the patterns observed in this model.

**Table 4:** Hypothesis testing.

Hypothesis	Path	*β*	SD	*t*	*p*	Conclusion
H1	MPSE → FL	0.093	0.028	3.252	0.001	Supported
H2	MPSE → MPA	−0.505	0.022	23.239	<0.001	Supported
H3	MPSE → MPWB	0.404	0.018	22.065	<0.001	Supported
H4	FL → MPA	−0.075	0.026	2.874	0.004	Supported
H5	FL → MPWB	0.182	0.018	10.139	<0.001	Supported
H6	MPA → MPWB	−0.500	0.018	28.045	<0.001	Supported

Table 5 presents the indirect effects with 95% bias-corrected bootstrap confidence intervals based on 5,000 subsamples. The results indicate that MPSE was indirectly associated with MPA via FL (*β* = −0.007, 95% CI [−0.013, −0.002]) and with MPWB via FL (*β* = 0.017, 95% CI [0.005, 0.026]). Additionally, MPSE was indirectly associated with MPWB via MPA (*β* = 0.252, 95% CI [0.225, 0.279]), while the indirect association involving both FL and MPA was statistically significant but very small in magnitude (*β* = 0.003, 95% CI [0.001, 0.007]). Finally, FL was indirectly associated with MPWB via MPA (*β* = 0.037, 95% CI [0.011, 0.061]).

**Table 5:** Indirect effects (95% bias-corrected bootstrapping).

Path	*β*	SD	*t*	*p*	Bias	2.5%	97.5%
MPSE → FL → MPA	−0.007	0.003	2.207	0.027	0.000	−0.013	−0.002
MPSE → FL → MPWB	0.017	0.005	3.241	0.001	0.001	0.005	0.026
MPSE → MPA → MPWB	0.252	0.014	18.626	<0.001	0.000	0.225	0.279
MPSE → FL → MPA → MPWB	0.003	0.002	2.214	0.027	0.000	0.001	0.007
FL → MPA → MPWB	0.037	0.013	2.876	0.004	0.001	0.011	0.061

**Figure 2:**
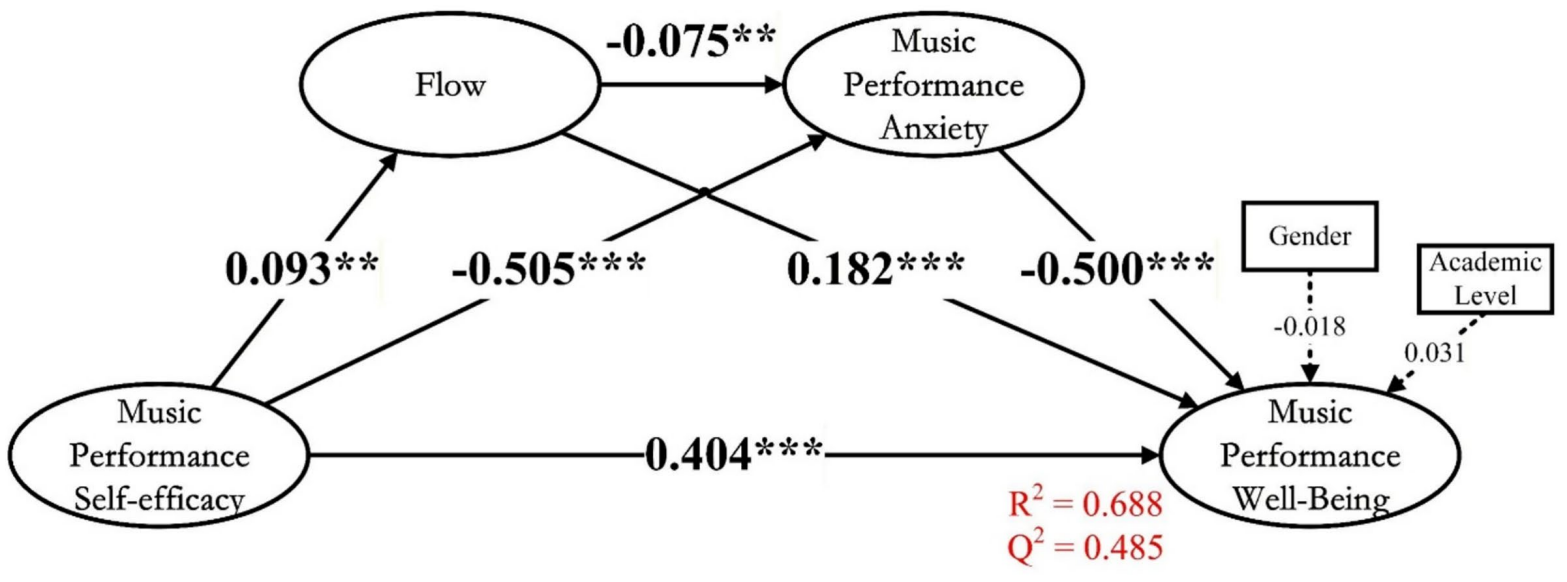
PLS results.

The *R*^2^ value for MPWB was 0.688, indicating that the model explained 68.8% of the variance in MPWB. In addition, the *Q*^2^ value for MPWB was 0.485, which is well above zero, demonstrating strong predictive relevance of the model. Effect sizes (*f*^2^) were further examined to assess the relative contribution of each predictor to MPWB. When MPWB was treated as the endogenous variable, MPA (*f*^2^ = 0.585) and MPSE (*f*^2^ = 0.385) showed a large effect size. FL (*f*^2^ = 0.104) demonstrated a small effect size. Together with the direct and indirect associations reported above, these results suggest that the FL-related associations were statistically significant but relatively modest, whereas MPSE and MPA showed stronger associations with MPWB. Figure 2 illustrates the results of the PLS-SEM analysis.

Supplementary analyses provide a stepwise comparison between the original model and the alternative model (MPSE → MPA → FL → MPWB) (Supplementary Tables S1–S5). First, in the alternative model, the path from MPSE to FL was not statistically significant (Supplementary Table S1), whereas other paths exhibited minor differences compared with the original model. Notably, the associations between FL and MPA remained statistically significant regardless of the ordering. Second, in the mediation analyses (Supplementary Table S2), all indirect associations remained statistically significant in the alternative model, although their magnitudes varied slightly compared with the original model. Third, comparisons of *R*^2^ (Supplementary Table S3) show similar overall variance explained for MPWB, despite minor differences in FL and MPA. Fourth, effect size comparisons (Supplementary Table S4) reveal slight shifts in relative contributions of FL and MPA across the models. Finally, model fit indices (Supplementary Table S5) are comparable, confirming that both model specifications are statistically feasible. Together, these results indicate that the alternative ordering primarily changes the pattern and magnitude of specific associations, especially those involving FL, while the overall model structure and the total variance explained for performance well-being remain largely similar. These findings suggest that the ordering between FL and MPA should be interpreted as a theoretically specified model arrangement rather than as evidence of temporal or causal precedence.

The measurement invariance across groups was tested using the MICOM procedure before the multi-group analysis. To ensure consistency in the baseline for comparison, group 1 and group 2 shared the same indicators, construct operationalizations, data handling procedures, and algorithm settings, as was done in Step 1 to confirm the configural invariance. Permutation tests were used to assess the compositional invariance in Step 2. As revealed in the results, all the original correlations were above the 5% thresholds of the quantiles, and the corresponding permutation *p*-values were above the 0.05 threshold, which is the statistical threshold for accepting the absence of compositional invariance between groups. The equality of the mean values and the variances between the groups was assessed in Step 3. The findings reveal that some constructs failed to meet the full invariance criterion, as their mean- or variance-based permutation p-values were less than 0.05 (FL and MPWB). Thus, full invariance was not achieved. Partial measurement invariance is verified, however, because Steps 1 and 2 were successfully completed, multi-group comparisons of path coefficients are valid, and note that there are group differences in means and variances (Henseler et al., 2016).

**Figure 3:**
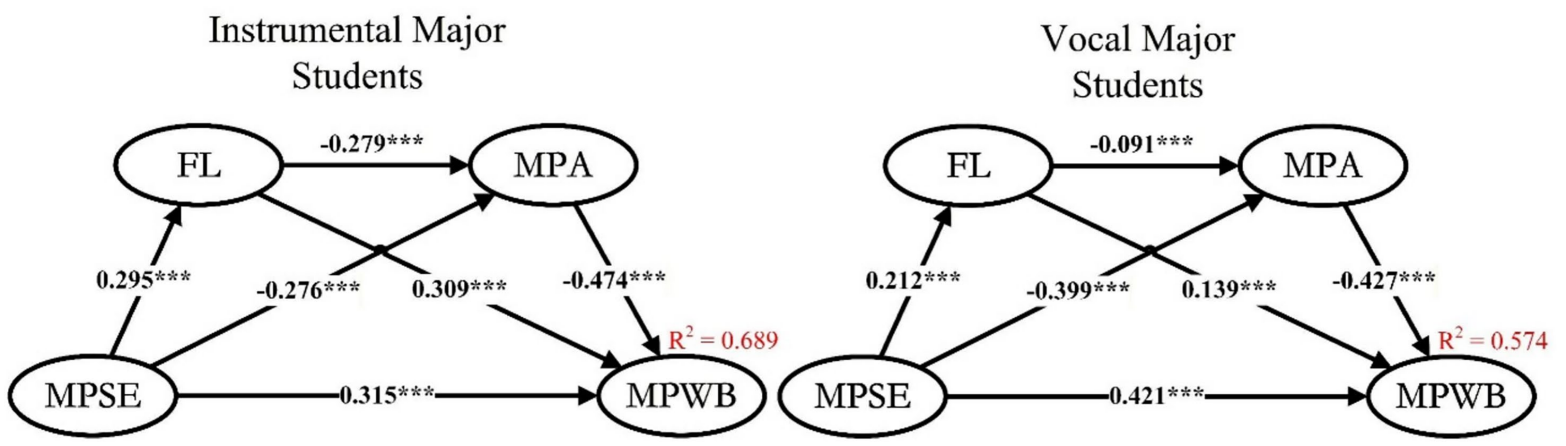
PLS results for vocal and instrumental music students.

Partial measurement invariance was derived with the MICOM procedure and then Henseler’s permutation-based multi-group analysis was performed to test for potential differences in path coefficients of the vocal and instrumental groups. Table 6 reveals significant differences between instrumental music students and vocal music students on several paths of the multi-group analysis. There are differences in the associations between the groups, with relatively stronger associations for vocal music group for the relationships between MPSE to MPA (Δ*β* = 0.123), MPSE to MPWB (Δ*β* = −0.105), and FL to MPA (Δ*β* = −0.188). In contrast, the correlation between FL and MPWB in the instrumental music group is relatively high (Δ*β* = 0.169). There was no significant difference between the two groups in the paths between MPSE to FL or MPA to MPWB. The results of the PLS path model for the vocal and instrumental samples are shown in Figure 3.

**Table 6:** Multi-group analysis (instrumental music − vocal music).

Path	Instrumental music		Vocal music		Instrumental − vocal	
	*β*	*p*	*β*	*p*	Δ*β*	*p*
MPSE → FL	0.295	<0.001	0.212	<0.001	0.083	0.130
MPSE → MPA	−0.276	<0.001	−0.399	<0.001	0.123	0.003
MPSE → MPWB	0.315	<0.001	0.421	<0.001	−0.105	0.003
FL → MPA	−0.279	<0.001	−0.091	0.037	−0.188	0.001
FL → MPWB	0.309	<0.001	0.139	<0.001	0.169	<0.001
MPA → MPWB	−0.474	<0.001	−0.427	<0.001	−0.048	0.197

## 5 Discussion and implications

This study introduces and empirically examines “MPWB” as a context-specific psychological well-being construct. MPWB conceptually differs from traditional subjective well-being, which has been the primary focus of previous research, as it represents a context-specific experience of well-being generated during musical performance rather than general life satisfaction. All variables included in the model are directly derived from the musical performance context, allowing assessment of their associations with well-being within performance settings. The results further indicate that MPWB is associated with multiple psychological variables within a multi-path model, highlighting the complexity of performance-related well-being. This underscores the theoretical value of adopting an interconnected perspective in the psychology of musical performance when examining performers’ psychological states and well-being during performance. It is worth noting that the relative statistical contributions of the proposed pathways were not equivalent. Both the direct and indirect associations involving FL were statistically significant but relatively small in magnitude, whereas MPSE and MPA showed more prominent associations with MPWB. It should also be emphasized that, because the data were collected at a single time point, the proposed ordering among MPSE, FL, MPA, and MPWB should be understood as a theoretically specified pattern of associations rather than an empirically verified causal sequence. The proposed model accounts for approximately 69% of the variance in MPWB and demonstrates strong predictive relevance, indicating both its explanatory and predictive value within the theoretical network and providing preliminary empirical support for positioning MPWB as a context-specific psychological construct. Overall, this study not only situates well-being explicitly within the music performance context, thereby extending the scope of traditional well-being research, but also offers a systematic theoretical example for future investigations of complex psychological associations in musical performance. The multi-group analysis indicates that the associations among MPSE, FL, MPA, and MPWB vary between vocal and instrumental students. Differences are observed in the configuration and relative weights of key paths, with some paths differing significantly across groups while others show no significant differences, highlighting domain-specific variations in the observed psychological associations. These findings suggest that models in the psychology of musical performance should consider performance-type specificity rather than assuming uniform psychological patterns across all musical contexts. Based on these observed associations, we next turn to a detailed examination of the individual psychological variables and their interrelations within the proposed theoretical framework.

MPSE was significantly associated with FL, MPA, and MPWB, and significant indirect associations involving FL and MPA were observed in relation to MPWB. In line with these results, González et al. (2018) and Spahn et al. (2021) found that higher self-efficacy was associated with lower performance anxiety and greater performance engagement among musicians. This pattern also aligns with the view that musical performance is related not only to technical proficiency, but also to performers’ task engagement and responses to performance-related emotional demands (Sloboda, 2000). From the perspective of social cognitive theory, this may be related to the role of self-efficacy in how performers judge their ability to cope with demanding situations (Bandura, 1986, 1997). More specifically, self-efficacy not only reflects individuals’ beliefs about their own capabilities, but is also closely related to their cognitive appraisal, emotional responses, and behavioral choices in high-demand contexts. In music performance, students with stronger MPSE may be more likely to perceive their performance capabilities positively and experience performance tasks as manageable, controllable, and less threatening. This may help interpret the observed associations between higher MPSE, lower MPA, and more positive MPWB experiences. Notably, although higher MPSE was also associated with higher FL, the relatively small coefficient and limited indirect association suggest that FL should be interpreted as a supplementary experiential correlate, while the MPA-related indirect association appears more statistically prominent. One possible interpretation is that FL may be more closely related to immediate situational conditions, such as challenge-skill balance and highly focused attention, than to relatively stable self-efficacy beliefs alone (Csikszentmihalyi, 1990; Fong et al., 2015). In teaching practice, educators may consider providing students with progressive and manageable performance opportunities, such as classroom demonstrations, studio recitals, and public performances, which may allow students to accumulate successful performance experiences. Teachers may also give task-focused feedback on technical execution, musical expression, and stage preparation, helping students recognize specific areas of competence rather than focusing only on errors or final outcomes. Such practices are consistent with the observed associations among students’ performance-related confidence, lower performance anxiety, and higher music performance well-being in the present study.

Multi-group analysis indicated that the associations between MPSE and MPA and between MPSE and MPWB differ significantly between instrumental and vocal students, with the associations being stronger for vocal students. This pattern may be interpreted from the perspective of embodied cognition. In vocal performance, the body itself functions as the primary medium of sound production, and performers’ voice, facial expressions, breathing, posture, and bodily sensations are directly involved in performance and exposed to audience evaluation (Kenny, 2011; Steptoe and Fidler, 1987). Such embodied exposure may intensify self-awareness, self-monitoring, and sensitivity to external judgment, making performance-related confidence particularly relevant to vocal students’ anxiety and well-being experiences. The stronger associations of MPSE with MPA and MPWB among vocal students may therefore be understood as reflecting the relevance of perceived capability in relation to bodily exposure and evaluative pressure during singing. By contrast, the association between MPSE and FL did not differ significantly between vocal and instrumental students. One possible interpretation is that flow is closely related to task-skill balance, focused attention, and accumulated practice, which are important conditions for both vocal and instrumental performance. These findings suggest several practical implications for differentiated music instruction. For vocal students, because the associations of MPSE with MPA and MPWB were stronger, teachers may place greater emphasis on building confidence in evaluative performance settings. For example, vocal teachers may use gradual exposure to peer singing, studio recitals, and examination-like performances, while providing feedback on vocal expression, stage presence, breathing control, and performance preparation. For instrumental students, confidence-related support remains relevant, but teaching may place greater emphasis on students’ sense of control over technical execution and performance preparation, such as through staged technical run-throughs, mock performances, and feedback on accuracy, coordination, and expressive control. For both groups, tasks with clear goals and appropriate challenge levels may help students perceive performance demands as manageable and matched to their current skills.

FL was found to be positively associated with MPWB and negatively associated with MPA. This pattern may be interpreted through flow theory, which regards flow as a deeply absorbed and task-focused state characterized by concentrated attention, reduced self-consciousness, a sense of control, smooth involvement, and intrinsic enjoyment (Csikszentmihalyi, 1990, 2000). These characteristics may be related to MPWB because they reflect positive affective experiences, psychological adaptation, and functional engagement during music performance. At the same time, when performers are immersed in the musical task, their attention may be directed more toward sound production, musical expression, and performance continuity than toward mistakes, external evaluation, or fear of failure (Kenny, 2011; Spahn et al., 2021). Nevertheless, although FL was significantly associated with MPA and MPWB, its effect size and association magnitudes were relatively small compared with those of MPSE and MPA. Thus, FL remains theoretically meaningful, but it should be understood as a supplementary experiential correlate rather than a primary explanatory factor for MPWB. One possible interpretation is that FL is a highly contextual and process-oriented state. Flow theory emphasizes the importance of the dynamic fit between task demands and personal skills, as well as clear goals, focused attention, and immediate feedback (Csikszentmihalyi, 1990; Schiepe-Tiska and Engeser, 2012). In this sense, FL in music performance may mainly reflect performers’ momentary immersion and in-task experience quality, rather than a stable factor strongly associated with overall MPWB. It is important to note that, although the present study models FL as being negatively associated with MPA and finds a significant relationship, this association should not be interpreted as a simple unidirectional causal effect. As Spahn et al. (2021) highlighted, pre-performance anxiety may be associated with difficulty entering flow, whereas performers’ immersion in the task can coincide with heightened flow experiences during performance. In the current study, we focus specifically on performers’ states during performance, which provides a theoretical rationale for specifying the “FL → MPA” association in this order in the proposed model. Nonetheless, the interplay between flow and anxiety may vary across different stages of the performance, and the strength and direction of their association could change over time. Consequently, the “FL → MPA” association observed in this study should be interpreted as a theoretically specified association within the proposed model rather than evidence of temporal or causal precedence. Future research could adopt longitudinal or experience-sampling designs to examine the potential reciprocal dynamics between flow and performance-related anxiety across different stages of music performance, from pre-performance anticipation to post-performance reflection. Given the relatively modest contribution of FL, flow-supportive strategies should be regarded as complementary rather than primary pedagogical approaches. Educators may support task-focused engagement by setting clear musical goals, encouraging complete run-throughs, and guiding students’ attention toward sound quality, musical structure, and expressive meaning. However, these practices should be integrated with broader confidence-building and anxiety-regulation strategies, which are more consistent with the stronger associations observed in the present findings.

Although FL showed a relatively modest contribution in the full sample, the multi-group analysis indicated significant differences between instrumental and vocal students in the associations of FL with MPA and MPWB. Specifically, the negative association between FL and MPA and the positive association between FL and MPWB were stronger among instrumental students than among vocal students. These results suggest that the psychological relevance of FL may vary according to performance modality. From the perspective of embodied cognition, instrumental performance involves the dynamic integration of the body and the instrument, in which performers coordinate motor control, auditory feedback, tactile perception, and technical execution in real time (Watson, 2006; Luciani et al., 2022). In this context, flow-related immersion may be more closely associated with performance continuity, technical control, and auditory-motor coordination, which may help interpret why FL showed stronger associations with MPA and MPWB among instrumental students. By contrast, vocal performance relies on the body itself as the sound-producing medium, involving breathing, resonance, articulation, facial expression, posture, and bodily self-awareness (Burwell, 2006; Heisel, 2015). Vocal students’ performance experiences may also be related to internal bodily feedback and evaluative exposure, such as self-monitoring and sensitivity to external judgment (Papageorgi et al., 2013; Wells, 1995). This may help explain why the associations of FL with anxiety and well-being were relatively weaker among vocal students than among instrumental students. These findings suggest that flow-related pedagogical support may be considered differently according to performance type, but such support should remain complementary to broader strategies targeting confidence and anxiety regulation. For instrumental students, teachers may consider incorporating task-immersion strategies during technically demanding passages, as FL showed stronger associations with MPA and MPWB in this group than in the vocal group. In practice, this may involve complete run-throughs that draw attention to auditory-motor coordination, phrase continuity, and expressive control, rather than focusing only on isolated errors. For vocal students, FL remains relevant, but teachers may need to consider the additional bodily and expressive demands of singing, such as breathing, resonance, articulation, facial expression, and stage presence. Thus, vocal training may integrate task-engagement activities with attention to bodily awareness and expressive regulation.

The path from MPA to MPWB exhibited the largest coefficient, indicating that higher anxiety levels were strongly associated with lower well-being among musicians, which is consistent with previous findings (Yang et al., 2025; Hernández-Dionis et al., 2025). This finding can be interpreted through Gross’s theory of emotion regulation, which emphasizes that emotional responses are shaped by situation appraisal, attentional deployment, cognitive reappraisal, and response modulation rather than by external events alone (Gross, 1998, 2015). In music performance contexts, MPA reflects not only nervousness, but also performers’ appraisal of the performance situation as threatening, including concerns about mistakes, failure, negative evaluation, and uncontrollable outcomes (Kenny, 2011). Higher MPA may be associated with greater self-monitoring, fear of errors, and evaluative concerns, as well as less psychological availability for musical expression, performance engagement, and positive emotional experience. This may help interpret the strong negative association between MPA and MPWB observed in the present study. Moreover, multi-group analysis indicated that the association between MPA and MPWB did not differ significantly between instrumental and vocal students. This robust negative relationship may reflect a general pattern in which higher anxiety is associated with greater attentional and cognitive load, stronger self-monitoring, and greater concern over errors or external evaluation, all of which are closely related to lower subjective well-being during performance. These findings suggest that anxiety-related support may be relevant for both vocal and instrumental students. In teaching practice, educators may help students establish brief and stable pre-performance routines, such as centering exercises, physical relaxation, and mental preparation before examinations, recitals, or stage performances. Students may also be guided to recognize common anxiety-related cues, including excessive self-monitoring, fear of mistakes, and concern about external evaluation. After performances, teachers may encourage students to reflect on how they responded under pressure, focusing on manageable aspects of the performance rather than only on errors or final outcomes. Such practices are consistent with the strong negative association observed between MPA and MPWB.

Supplementary analyses (Supplementary Tables S1–S5) comparing the original model with a theoretically plausible alternative model (MPSE → MPA → FL → MPWB) showed that reordering FL and MPA was mainly associated with changes in associations involving FL. In particular, the path from MPSE to FL was no longer statistically significant, and the magnitudes of indirect associations involving FL were altered, although the association between FL and MPA remained significant. From a musical perspective, these differences may reflect the close interrelation between performers’ immersion in the music and moment-to-moment anxiety. When anxiety is positioned before flow in the model, the shared variance between MPA and FL may be associated with a weaker apparent relationship between MPSE and FL. In other words, the way performers experience flow and anxiety during performance may be interdependent, and association magnitudes can vary depending on the order in which these states are modeled, without implying temporal or causal precedence. This, however, illustrates a limitation of the current cross-sectional design, which does not allow determination of the directionality between flow and music performance anxiety. Future research could adopt longitudinal or experience-sampling designs to examine the potential reciprocal dynamics between flow and performance-related anxiety across different stages of music performance.

In addition to the discussion of the primary variables in the proposed model, the potential role of the control variables also warrants further consideration. In Chinese higher music education, female students generally constitute a larger proportion of students across many music-related disciplines. Although gender was included as a control variable in the present study and showed no significant direct association with MPWB, the highly feminized sample may still be relevant to the interpretation of path relationships involving performance anxiety and emotional experiences, as anxiety regulation and well-being experiences may vary across gender groups in music performance contexts. Therefore, the relationships among MPSE, FL, MPA, and MPWB should be interpreted with appropriate caution. Future studies are encouraged to recruit more gender-balanced samples and further examine potential gender differences through multi-group analyses. Similarly, although academic levels did not exhibit a significant direct association with MPWB, students at different stages of musical training may possess varying levels of performance experience, stage exposure, and psychological maturity. These differences may be associated with variations in how students experience performance pressure, regulate emotions, and respond to evaluative performance environments. Future research could further explore whether the structural relationships among the proposed variables differ between undergraduate and graduate student populations.

## 6 Limitations

First, the cross-sectional design of this study limits the ability to draw causal inferences and to determine the directionality of the observed relationships, particularly the ordering between FL and MPA. Given the relatively modest FL-related associations, the role of FL should also be interpreted cautiously and should be further validated through longitudinal or experience-sampling research. Future research is recommended to examine the temporal and directional dynamics among the key psychological variables. Second, the sample of Chinese university music students may not fully represent the global population of music students, which could limit the generalizability of the findings. Future studies with larger and more culturally diverse samples would help enhance the generalizability of the results. Third, self-reported information can be subject to social desirability or self-reported errors. In future studies, objective measures, behavioral observations, or multi-informant assessments could be added to self-report data and further minimize potential bias. Forth, the MPWB scale in this study remains an adaptation of the WHO-5 Questionnaire and does not represent a fully developed instrument specifically designed to measure well-being in the context of music performance. Although the adapted scale captures key performance-specific experiences, it may not cover all aspects of well-being relevant to musicians. Future research should aim to develop a dedicated MPWB scale using rigorous psychometric methods, such as exploratory factor analysis and confirmatory factor analysis, to ensure comprehensive construct coverage, structural validity, and cross-cultural applicability. Last, although the present study controlled for gender and academic level, other individual and training related factors, such as years of musical training and prior stage experience, as well as practice intensity and personality traits, were not included in the model. These variables may have affected the magnitude or direction of the observed relationships in the performance context, and were not included in the model. Future studies are encouraged to include these theoretically important covariates in order to better explore the psychological process of music performance and improve model’s explanatory power.

## Data Availability

The raw data supporting the conclusions of this article will be made available by the authors, without undue reservation.
