# Supplementary material for: On stage: the association between self-efficacy, flow, anxiety, and performers’ well-being in musical performance
Source: Front Psychol. 2026 Jul 8;17:1859921. doi: 10.3389/fpsyg.2026.1859921 (PMC13388851; doi:10.3389/fpsyg.2026.1859921)
Supplement: Supplementary file 1 [file Supplementary_File_1.docx]

**Supplemental Material**

Table S1. MPWB Scale

| Items | Description |
| --- | --- |
| MPWB1 | During music performance, I have felt cheerful and engaged with the music. |
| MPWB2 | During music performance, I have felt calm and composed. |
| MPWB3 | During music performance, I have felt energetic, focused, and fully involved. |
| MPWB4 | During music performance, I felt refreshed and focused. |
| MPWB5 | During music performance, I focused on the musical process and derived enjoyment from it. |

Table S2. Comparison of Direct Path Coefficients Between Original and Alternative Models

| Path | *β* | *SD* | *t* | *p* | Conclusion |
| --- | --- | --- | --- | --- | --- |
| MPSE → FL (Original Model) | 0.093 | 0.028 | 3.252 | 0.001 | Supported |
| MPSE → FL (Alternative Model) | 0.038 | 0.036 | 1.042 | 0.298 | Not-Supported |
| MPSE → MPA (Original Model) | -0.505 | 0.022 | 23.239 | < 0.001 | Supported |
| MPSE → MPA (Alternative Model) | -0.511 | 0.022 | 23.52 | < 0.001 | Supported |
| MPSE → MPWB (Original Model) | 0.404 | 0.018 | 22.065 | < 0.001 | Supported |
| MPSE → MPWB (Alternative Model) | 0.404 | 0.018 | 22.118 | < 0.001 | Supported |
| FL → MPA (Original Model) | -0.075 | 0.026 | 2.874 | 0.004 | Supported |
| MPA → FL (Alternative Model) | -0.097 | 0.035 | 2.777 | 0.006 | Supported |
| FL → MPWB (Original Model) | 0.182 | 0.018 | 10.139 | < 0.001 | Supported |
| FL → MPWB (Alternative Model) | 0.181 | 0.018 | 10.152 | < 0.001 | Supported |
| MPA → MPWB (Original Model) | -0.500 | 0.018 | 28.045 | < 0.001 | Supported |
| MPA → MPWB (Alternative Model) | -0.501 | 0.018 | 28.206 | < 0.001 | Supported |

Table S2. Mediation Analysis: Original vs. Alternative Model (95% bias-corrected bootstrapping)

| Path | *β* | *SD* | *t* | *p* | Bias | 2.50% | 97.50% |
| --- | --- | --- | --- | --- | --- | --- | --- |
| MPSE → FL → MPA | -0.007 | 0.003 | 2.207 | 0.027 | 0 | -0.013 | -0.002 |
| MPSE → MPA → FL | 0.050 | 0.018 | 2.738 | 0.006 | 0.001 | 0.013 | 0.084 |
| MPSE → FL → MPWB | 0.017 | 0.005 | 3.241 | 0.001 | 0.001 | 0.005 | 0.026 |
| MPSE → FL → MPWB | 0.018 | 0.006 | 2.764 | 0.006 | 0 | 0.007 | 0.020 |
| MPSE → MPA → MPWB | 0.252 | 0.014 | 18.626 | < 0.001 | 0 | 0.225 | 0.279 |
| MPSE → MPA → MPWB | 0.256 | 0.014 | 18.856 | < 0.001 | 0 | 0.229 | 0.282 |
| MPSE → FL → MPA → MPWB | 0.003 | 0.002 | 2.214 | 0.027 | 0 | 0.001 | 0.007 |
| MPSE → MPA → FL → MPWB | 0.009 | 0.003 | 2.732 | 0.006 | 0 | 0.003 | 0.016 |
| FL → MPA → MPWB | 0.037 | 0.013 | 2.876 | 0.004 | 0.001 | 0.011 | 0.061 |
| MPA → FL → MPWB | -0.018 | 0.006 | 2.764 | 0.006 | 0 | -0.030 | 0.005 |

Table S3. *R²* Comparison Between Original and Alternative Models

| Constructs | Original Model | Alternative Model |
| --- | --- | --- |
| FL | 0.009 | 0.013 |
| MPA | 0.267 | 0.261 |
| MPWB | 0.688 | 0.687 |

Table S4. *f²* Comparison Between Original and Alternative Models

| Constructs | Original Model | | | Alternative Model | | |
| --- | --- | --- | --- | --- | --- | --- |
|  | FL | MPA | MPWB | FL | MPA | MPWB |
| FL |  | 0.008 | 0.104 |  |  | 0.104 |
| MPA |  |  | 0.585 | 0.007 |  | 0.590 |
| MPSE | 0.009 | 0.344 | 0.385 | 0.001 | 0.354 | 0.386 |

Table S5. Model Fit Index Comparison Between Original and Alternative Models

| Model Fit Index | Original Model | Alternative Model |
| --- | --- | --- |
| SRMR | 0.061 | 0.064 |
| d_ULS | 1.738 | 1.675 |
| d_G | 0.249 | 0.235 |
| NFI | 0.907 | 0.912 |
